# Peer (dyadic) support: a hypertension feasibility study for older African American women

**DOI:** 10.3934/publichealth.2024048

**Published:** 2024-08-21

**Authors:** Angela Groves, Wesley Browning

**Affiliations:** 1 Bronson School of Nursing, Western Michigan University, Kalamazoo, MI 49008, USA; 2 Department of Research, Cizik School of Nursing at UT Health Houston, 6901 Bertner Ave, 5^th^ floor Houston, TX 77030, USA

**Keywords:** African Americans, women, hypertension, low sodium, DASH diet, peer (dyadic) support, peer support

## Abstract

**Introduction:**

African American women have a higher prevalence of hypertension than women of other ethnicities. The increasing prevalence of hypertension among this population is alarming.

**Methods:**

This was an 8-week feasibility study. The study population consisted of African American women aged 60 years and older with a self-reported diagnosis of hypertension. Participants identified a peer to participate with or were paired with another participant in the study. Educational sessions on communication, the Dietary Approaches to Stop Hypertension (DASH) diet, and home blood pressure monitoring were provided for participants. Participants were required to measure their blood pressure twice daily using an Omron monitor and communicate with their peers at least twice weekly. Dietary intake was measured pre- and post-intervention using the DASH Quality (DASH-Q) survey, social support was measured using the Medical Outcomes Study (MOS) Social Support Survey, and communication was tracked using communication logs. Feasibility was assessed by enrollment and retention rates.

**Results:**

Pre-intervention, participants had an average DASH-Q score of 33.76 (SD = 13.37). Participants' post-intervention DASH-Q scores increased by 5 points compared to their pre-intervention scores; however, this difference was not significant (t = −1.608, p = 0.059). Additionally, participants who completed the intervention had a 4-point decrease in their systolic blood pressure at week 6. A dependent sample t-test revealed the difference was significant (t = 2.305, p = 0.014). A total of 40 participants were enrolled in the study, and the retention rate was 85%.

**Conclusion:**

Although not statistically significant, improvements in systolic blood pressure and DASH diet adherence were observed. Therefore, the results indicate that the peer (dyadic) support intervention was feasible.

## 1. Introduction

In 2018, more African American women died from complications attributed to high blood pressure (37.5%) than Hispanic women (16.7%), Asian women (14.9%), and Caucasian women (19.6%) [1]. These data demonstrate that African American women are disproportionately affected by hypertension. African American women's eating habits and patterns put them at risk for chronic diseases [2],[3]. Maintaining healthy eating habits using the Dietary Approaches to Stop Hypertension (DASH) diet plan has reduced systolic blood pressure by 11 mmHg among individuals with hypertension [4]. To illustrate that, Jurascheck et al. [5] (2020) conducted a study showcasing the innovative application of the DASH diet in blood pressure management. Their research compared the DASH diet across varying sodium intake levels to investigate its potential for reducing blood pressure. Over half of the participants were black women, with an average age of 48 years. Results demonstrated that reducing sodium intake effectively lowers blood pressure. While the study excluded individuals with cardiovascular disease or those on antihypertensive medication, its findings strengthen the case for the DASH diet's efficacy in blood pressure reduction.

However, researchers report that African Americans, especially women, struggle to make the necessary dietary modifications to help control their blood pressure due to challenges that include the following: a general lack of low-sodium dietary knowledge, income barriers, limited food availability in neighborhoods, a preference for traditional family and alternative food practices that typically involve foods that are high in sodium, a lack of motivation to follow a low-sodium diet, and a lack of family support when preparing healthy, low-sodium meals [2],[3],[6].

Effective interventions and daily dietary practices must increase African American women's adherence to low-sodium diets [2],[3]. Existing studies have shown that African American women are aware that hypertension is a severe condition and attempt to change their eating habits by increasing their intake of fruits and vegetables; however, despite this awareness, they continue to consume unhealthy food [2],[3]. Hypertension treatment approaches are not always practical for African American women, and medication alone may not help lower their blood pressure. Although other lifestyle behavior changes can reduce blood pressure, dietary changes are one of the primary factors that positively improve blood pressure measurements in this group. The prevalence of hypertension among African American women has not diminished sufficiently despite the provision of low-sodium diet education and diet modification guidelines. Adopting low-sodium dietary practices presents a challenge for this group of women [2],[3].

Peer (dyadic) support comprises one or more individuals willing to hold each other accountable. It requires an individual who can be nonjudgmental and grounded in their approach to helping someone manage their health condition [7]. Importantly, researchers have indicated that social relationships may affect health behaviors. For example, Goldstein et al. [8] conducted a 12-week study to assess the feasibility and acceptability of peer partner support and coaching to provide health-related assistance and resources for veterans with chronic health issues. The study's findings revealed that participants highly valued the sense of accountability, encouragement, and ongoing support provided by their peer partners. Moreover, participants appreciated receiving text-based reminders to engage in dyadic communication with their peers and found group discussions with their partner and coach beneficial. However, participants noted that the effectiveness of peer support and coaching could be more consistent. They expressed a desire for more proactive self-assessment to identify when additional support, such as coaching, might be necessary.

Similar to the Goldstein et al. [8] study, Conley et al. [9] undertook a nine-week study to evaluate the effectiveness of dyadic support in promoting healthy eating and physical activity among African American church members. The findings revealed that offering encouragement and emotional support proved advantageous for those engaged in peer dyadic relationships. Additionally, participants found personal motivation in witnessing their partners' progress to improve their healthy eating habits and physical activity levels. Moreover, the majority expressed willingness to provide encouragement and reminders for goal setting if their peer partners struggled to achieve their goals. While neither study specifically targeted African American women, the noteworthy effectiveness of utilizing peer dyadic support is evident.

The focus and purpose of this study was to determine the feasibility of a dyadic (peer) support intervention for older African American women with hypertension. Feasibility studies are utilized to ascertain the suitability and viability of an intervention for further testing [10]. Because there is a dearth of published research combining the DASH diet and peer (dyadic) support to enhance blood pressure management among older African American women with hypertension, this study's goal is to assess the feasibility of such an intervention for future studies.

There is a critical need to develop successful interventions that will effectively improve low-sodium diet adherence and blood pressure management among African American women with hypertension. We hypothesized that the peer (dyadic) support intervention would be feasible, the participants' diets would improve, and there would be a reduction in systolic blood pressure.

## 2. Materials and methods

### 2.1. Instruments

Data on demographic characteristics were obtained using a 15-item questionnaire developed by the principal investigator. Participants were asked about their age, marital status, education level, and income.

The DASH Quality (DASH-Q) survey (outcome variable) assessed participant adherence to the DASH diet. The 11-item scale measures the consumption of DASH foods in the past seven days. Scores are summed. The DASH-Q has a sufficient internal consistency of 0.77−0.83 [11]. The DASH-Q survey was valid and reliable (Cronbach α = 83) in a hypertension feasibility study that included African Americans [12]. All participants completed the DASH-Q survey at baseline and post-intervention.

The participants used blood pressure logs, developed by the American Heart Association [13], to record their blood pressure twice daily (am and pm) throughout the 8-week program. They obtained their blood pressure using the Omron home blood pressure monitoring device. The Omron home blood pressure device was used in this study and has been widely used in hypertension intervention studies [12],[14]. To test the impact of the intervention on blood pressure, an average systolic blood pressure score was calculated for the first and the sixth weeks of the intervention. The sixth week was chosen because most participants did not provide adequate blood pressure data past that point.

A communication log was used to record the frequency and type of communication between each dyad, and the dyads recorded weekly interactions in a communication log. The principal investigator developed the log based on previous studies that used communication logs to document interpersonal interactions between dyads [9],[15],[16]. The principal investigator modified the communication log to include specific peer (dyadic) support content for this study.

The Medical Outcomes Study (MOS) Social Support Survey is a 19-item scale that measures the availability of support [17],[18]. All participants completed the MOS post-intervention. Individual items are rated on a scale from 1 to 5, with 1 = none and 5 = all of the time. The average score of all 19 items was calculated to determine social support scores. Feasibility was measured by enrollment and retention rates. Also, feasibility was measured based on the frequency of communication and length of time of each communication encounter.

### 2.2. Methods

A one-hour DASH diet education session, a one-hour communication training session, and a one-hour home blood pressure training session were provided for all participants. The training was conducted in person or virtually, depending on the participants' preferences A registered dietitian conducted the DASH diet education session. The DASH education session included the following topics: the definition of high blood pressure, the DASH diet plan, reading labels, menu planning, and culturally tailored heart-healthy recipes.

A psychology graduate student conducted communication training. Each participant participated in a one-hour communication training session. The training session included the following topics: defining communication, stages of relationships, relationship dynamics, how to engage in conversation, understanding nonverbal messages, identifying the types of destructive and constructive communication, developing active and reflective listening skills, strategies to resolve conflict in communication, how to check in with another person, and how to document information in the communication logs [9],[15],[16]. All participants needed to communicate with their partner at least twice weekly, either by phone, email, in person, or virtually. Participants were provided with weekly topics to discuss with their peer partners. The weekly discussion topics aimed to facilitate communication about the DASH diet and blood pressure monitoring. See Table 1 for weekly discussion topics. In addition, a research assistant provided weekly newsletters by email to all participants. The purpose of the weekly newsletters was to reinforce the DASH diet, blood pressure, and communication training.

A registered nurse completed blood pressure training. Each participant participated in a one-hour home blood pressure monitoring training on proper techniques. Participants met with the principal investigator and research assistant, either in person or online, at baseline, one month, and postintervention to ensure the precision and accuracy of blood pressure measurements and to answer any questions or concerns.

Participants received a $20 Walmart gift card for completing the educational sessions and a $85 Walmart gift card for completing the intervention.

**Table 1.** Weekly discussion of topics.

Week	Topics
Week 1	Review the Dash Diet plan and identify 2–3 foods low in sodium. Discuss 2–3 diet and blood pressure self-management goals. Discuss reading food labels and portion control.
Week 2	Make a shopping list of 2–3 healthy low-sodium foods to substitute at the grocery store. Prepare and cook 1–2 meals using a DASH-friendly recipe.
Week 3	Discuss using herbs, spices, citrus juices, and vinegar instead of salt to add flavor to foods. Prepare and cook 1–2 meals using a DASH-friendly recipe.
Week 4	Prepare and cook 1–2 meals using a DASH-friendly recipe. Discuss strategies with the family for meal planning. Discuss progress toward achieving goals.
Week 5	Discuss strategies for eating a low-sodium meal at a restaurant. Discuss replacing 1–2 desserts and snacks with fruits and other foods low in saturated fat, trans fat, cholesterol, sodium, sugar, and calories.
Week 6	Cook one meal by grilling, roasting, braising, searing, or sautéing to bring out the natural flavor of the food and reduce the need to add salt when cooking.
Week 7	Unless contraindicated, discuss incorporating one potassium food in the diet, such as sweet potatoes and bananas. Prepare and cook 1–2 meals using a DASH-friendly recipe. Discuss strategies for family meal planning. Discuss progress toward achieving goals.
Week 8	Discuss overall progress toward achieving goals, including successes, challenges, and barriers.

### 2.3. Data analysis

We first calculated means, standard deviations, and frequencies to describe the sample. Then, to test the hypothesis that the intervention successfully improved DASH diet adherence, we utilized a dependent samples t-test for participants who completed the intervention (n = 34). To address the hypothesis that the intervention successfully improved blood pressure, we performed a similar dependent samples t-test for individuals who completed the intervention and had complete blood pressure data (n = 29) to determine whether blood pressure significantly increased over the eight weeks.

## 3. Results

### 3.1. Descriptive statistics

Complete descriptive statistics are provided in Table 2. Forty participants (20 dyads) were enrolled in the study. Of these participants, 34 (17 dyads) completed the intervention. Over 75% of the dyads opted for a partner, friend, or family member, while the researcher paired the remaining participants (25%). In the sample of participants who completed the intervention, the average age was approximately 71 years (M = 71.38 years, SD = 8.38). Seven participants were married (20.6%). Half of the participants had an income below $30,000. Of this group, two participants had missing BMI data; thus, the 32 participants who provided complete BMI data had an average BMI of 30.32 kg/m^2^ (SD = 5.21). Participants reported relatively high levels of social support post-intervention (M = 4.15, SD = 0.94). Pre-intervention, participants had an average DASH-Q score of 33.76 (SD = 13.37) and average systolic blood pressure of 130.06 (SD = 13.62).

**Table 2.** Descriptive statistics of participants who completed the intervention.

Variable	N = 34 Mean (std)/n (%)
Age	71.38 (8.38)
Married	7 (20.6%)
Education level
High school	11 (32.4%)
Some college	6 (17.6%)
Vocational school	1 (2.9%)
Associate's degree	6 (17.6%)
Bachelor's degree	2 (5.9%)
Master's degree	1 (2.9%)
Doctoral degree	4 (11.8%)
Grammar school	1 (2.9%)
Professional degree	2 (5.9%)
Under $30,000 yearly income	17 (50.0%)
BMI (n = 32)	30.34 (5.21)
Emotional/informational support	4.22 (0.91)
Tangible support	3.98 (1.31)
Affectionate support	4.32 (1.05)
Positive social interaction support	4.14 (1.06)
Total social support	4.15 (0.94)
Pre-intervention diet	33.76 (13.37)
Post-intervention diet	38.82 (13.38)
Week 1 AMS (n = 29)	130.06 (13.62)
Week 6 AMS (n = 29)	126.01 (12.92)

### 3.2. Intervention results

#### 3.2.1. Feasibility

Forty participants were enrolled in the study, and 34 (85%) completed the intervention. Participants' communication logs indicated that the dyads communicated an average of 2–3 days per week via phone call, in person, or by text. The length of time for each communication encounter lasted from 2 minutes to 2 hours.

Participants found the peer (dyadic) support intervention helpful, noting that it made them more mindful of their eating habits and blood pressure monitoring. (MF/MB) “This process has made me more mindful of looking at how much sodium intake I've been taking because I see the difference.” (J/MB) “I was more cognizant of taking my blood pressure.” The peer support system offered motivation and accountability, which the participants deemed acceptable. (B/MB) “For me, I think the accountability of a peer partner was a good part for me.” (W5/EC) “Being with a buddy really helps.”

#### 3.2.2. DASH adherence and blood pressure

Dependent samples t-tests were used to compare participants' baseline and post-intervention scores. On average, participants who completed the intervention had post-intervention DASH-Q scores approximately 5 points higher; however, this difference was not significant (t = −1.608, p = 0.059). On average, participants who completed the intervention had a 4-point decrease in their systolic blood pressure. The dependent samples t-test revealed that this difference was significant (t = 2.305, p = 0.014).

## 4. Discussion

The increasing prevalence of hypertension among African American women is alarming. One way to address health disparities among African American women is through a peer-support mechanism. Social support among peers can positively influence health behaviors through emotional support and cohesion [9]. Our study leveraged the importance of peer (dyadic) support to improve systolic blood pressure among older African American women. In particular, the participants received DASH diet, blood pressure, communication education, and guided opportunities to apply the content. Overall, the DASH intervention with dyadic (peer) support was feasible for this population. The retention rate in the current study was 85%, which is higher than the 54% enrollment rate and lower than the reported 100% retention rate reported in Wright et al. [12].

In the current study, participants on the DASH diet improved their intake of healthy food options. However, the findings were not statistically significant, possibly related to the smaller sample size due to attrition. Most importantly, participants who finished the intervention observed a decrease in systolic blood pressure levels compared to their initial readings. The reduction in systolic blood pressure and the increase in healthy food choices prove the intervention is efficacious in this population. Although the study did not show statistical significance in DASH diet improvement, the study aimed to determine feasibility. Therefore, the study showed that women on the DASH diet can improve their intake of healthy food options by adding the peer (dyadic) support mechanism. These findings are similar to those of the study conducted by Jurascheck et al. [5], which reported that foods consumed on the DASH diet decreased blood pressure.

Based on the MOS survey results, participants in this study reported significant levels of social support post-intervention. This outcome aligns with Byrd et al. [19], which observed a positive correlation between social support and reduced systolic blood pressure. However, neither study recorded participants' levels of social support prior to the intervention. It is important to note that both studies emphasized the role of social relationships in lowering blood pressure among African Americans.

Based on the current study's findings, potential strategies exist for enhancing feasibility. For instance, incorporating an assessment of intervention fidelity throughout the 8-week study period would have bolstered the robustness of the findings. Additionally, employing a tool to evaluate feasibility would have enhanced the rigor of assessment and analysis. Moreover, considering the 15% attrition rate, delving into the reasons behind participant non-completion of the intervention would have been beneficial. Understanding both participant motivations for participation and non-participation could have provided valuable insights. Lastly, critical points from the analysis highlighted the effectiveness of peer (dyadic) support in promoting adherence to the DASH diet and improving blood pressure management through mutual encouragement and accountability.

This study had strengths and limitations. It included the (dyadic) support mechanism, promoting health behavior changes such as improved DASH diet adherence and systolic blood pressure. Obtaining complete blood pressure data from participants was an issue, as there was missing data from blood pressure logs, especially during weeks 7 and 8. The MOS scale was only administered post-intervention; therefore, we need to determine if participants had adequate social support before the intervention. The sample size was small due to attrition; however, the current study's findings should be considered in the context of a small feasibility study. The sample was limited to older African American women, and therefore, we do not know the feasibility of this study for African American women aged less than 60 years with hypertension. It is essential to recognize that participants in the study were matched with familiar individuals such as family and friends or paired by the researcher. Consequently, we need insight into the extent of influence or effectiveness of communication within dyads who were already acquainted compared to those who were not. Moreover, this study did not evaluate participants' interest in the intervention, their motivations for participating, or their reasons for not completing the study, all of which are suited to qualitative research methods.

## 5. Conclusions

The findings in the current study indicate that (peer) dyadic support can help to improve the consumption of healthy foods and decrease systolic blood pressure among African American women with hypertension on the DASH diet. Future randomized controlled trials should be conducted to evaluate the effectiveness of the peer dyadic support modality on DASH diet adherence to reduce systolic blood pressure. Additionally, future longitudinal studies should be conducted to assess the effectiveness of peer (dyadic) support on DASH diet adherence to reduce systolic blood pressure over time in this population.

## Use of AI tools declaration

The authors declare they have not used Artificial Intelligence (AI) tools in the creation of this article.
